# The Effect of Tool Geometry on the Strength of FSW Aluminum Thin Sheets

**DOI:** 10.3390/ma15228187

**Published:** 2022-11-18

**Authors:** Achilles Vairis, Markos Petousis, Nikolaos Mountakis, Charikleia Tsarouchidou, Nectarios Vidakis

**Affiliations:** Mechanical Engineering Department, Hellenic Mediterranean University, 71410 Heraklion, Greece

**Keywords:** friction stir welding (FSW), tensile strength, aluminum Al 7075, welding speed, thin sheet, rotational speed, tool geometry

## Abstract

Welding tools of different designs have been used to join friction stir welding 2-mm-thick Al 7075 sheets, to investigate the effect of the tool geometry on the weld performance. Five cylindrical tools with different pin geometries were manufactured from heat-treatable low alloy steel WNr 1.6582/DIN 34CrNiMo6. Additionally, the effect of the welding speed was considered in the work, with six different speeds ranging from 80 mm/min to 300 mm/min. The weld tool rotational speed was kept constant at 1000 rpm and all other parameters were also kept constant in the experiments. The tensile strength was measured to investigate the mechanical properties of the weld. Results were processed with statistical analysis tools, which showed that the mechanical strength was affected by tool geometry as well as welding speed. The weld tool with the highest pin diameter achieved the highest tensile strength. The welding speed affected the tensile strength differently in the different weld tool geometries studied. The highest weld efficiency reported in the tests is 72.20%, achieved with a cylindrical pin weld tool at 250 mm/min.

## 1. Introduction

Over the last decades, aluminum alloys have been used as the material of choice for engineering components, as they possess desirable properties, such as an adequate weight-to-strength ratio. Such materials allow the replacement of steel, and they are being used more in industries where traditional fusion welding was the preferred procedure employed, such as aerospace, transportation, and marine industries. Fusion welding of aluminum parts can produce weld defects such as oxides, porosity, and hot cracking, which friction stir welding does not produce. Although this solid-state technique does not have portable welding machines, it is cheaper as it does not require non-destructive testing (NDT) checks to the same extent as fusion welded parts.

The solid-state friction stir welding (FSW) technique of aluminum alloys was invented 30 years ago and has been used extensively for joining aluminum alloy parts. The process was developed to address joining issues related to specific metals, which were difficult to weld [1]. The effects of welding parameters on aluminum alloy sheet joints have been studied for various Al series, such as AA5083 [2], AA1050 [3,4], AA6013 [5], and AA7075 [6,7]. Studies have expanded to dissimilar material joints (for example AA2024 with AA7075) [8], as well as, more recently, to polymeric materials, such as polypropylene (PP) [9] and polyamide 6 (PA6) [10,11,12], among others. In addition, research has also been undertaken on joining 3D printed polymeric materials, such as acrylonitrile butadiene styrene [13], polylactic acid (PLA) [14], and poly (methyl methacrylate) (PMMA) [15], but the literature remains limited at the moment.

According to Czerwinski et al. [16], a non-consumable tool with a protruding pin is made to traverse through the joint. Heat is produced because of the friction between the shoulder of the tool and the workpiece, which results in the material softening around the pin while producing microstructural changes in the welding zone [17]. As Dewangan et al. [18] commented, there are four zones in a typical FSW joint: nugget zone (NZ), thermomechanical affected zone (TMAZ), heat affected zone (HAZ), and base material (BM). The nugget zone is also called the stir zone and is located at the centerline of the weld, being the zone that is highly deformed and recrystallized. Kumar et al. [19] estimated that the heat generated during welding is equivalent to the tool’s power input to the weld.

For FSW to produce sound joints, appropriate process parameters have to be used. As Mishra et al. [20] identified, the welding parameters, tool geometry, and design of the joint have a significant effect on the material flow pattern and temperature distribution. These, as a result, affect the final microstructure of the material.

The aluminum alloy sheets used in this study are of the series 7xxx, where the main chemical element is zinc with a concentration of 1% to 8%. When magnesium is added in small quantities, the alloy becomes heat-treatable and its strength increases [21]. The 7075 alloy shows an average corrosion resistance or weldability compared to other alloys, while having a high strength at a low density of 2.81 g/cm^3^, and for that reason, it is used in ships, airplanes, etc. It is a hard alloy; thus, the hardness of the tools is important for successful welding, though geometry is also important. Terra et al. [22] found that for the selected process parameters and tool geometry, the maximum radial force occurred at 45° of the welding direction, while Lertora et al. [23] experimented with the spindle tilt angle that was fixed at 1°. Hattingh et al. [24] concluded that a higher taper angle is beneficial in producing better plastic flow conditions that are conducive to obtaining high-strength welds. Rao et al. [25] concluded that the main reasons for the defects in a weld are the shorter pin length, shoulder diameter, and insufficient heat generation. Elangovan et al. [26] identified that tools with square pin profiles are known to produce defect-free welds with superior tensile properties.

More welding experiments with various tool geometries, such as pentagonal, hexagonal, taper cylindrical (TC), taper cylindrical with thread (TCH), frustum (FPT), triangular (TPT), and cubic (CPT), have been conducted by Naik et al. [27] and Kumar et al. [28], who reported that joints made using the SQ tool pin profile possessed improved mechanical properties compared to other tool pin profiles. Patel et al. [29] identified that the tapper cylindrical pin is not suitable with a shoulder diameter of 18 mm for sound welds, meanwhile, Lammlein et al. [30] reported that the TC pin tool reduced the process forces remarkably. Ramanjaneyulu et al. [31] concluded that the rate of heat generation and peak temperatures reached are relatively higher in the case of non-circular pin profiles, increasing with the number of flats.

In comparison, Cetkin et al. [32] and Xu et al. [33] conducted similar experiments with a triangle-shaped tool. Moreover, Krishna et al. [34] investigated, in addition to pin shape, different tool diameters. Another interesting paper was by Chegeni et al. [35] involving the use of two tools to make a simultaneous double pass along the weld line, which proved that heat input through the material would otherwise be the case if a full penetration single pass weld was made.

Different welding tools have also been tested, such as underwater friction stir welding tool pin profiles, where Xu et al. [36] identified that when the pin has additional edges, a more intense internal plastic deformation develops, which leads to an improved internal material flow in stir zone (SZ), while surface material flows remained the same for all joints. Bahrami et al. [37] used optical microscopy (OM) and scanning electron microscopy (SEM) and measured the tensile strength to conclude that the highest UTS was obtained with a triangular pin geometry. Murthya et al. [38] used image processing and acoustic emission techniques to investigate the effect of the concave shoulder with a conical threaded pin tool geometry.

Although aluminum alloys have been studied extensively, research on the 7075 alloys is still limited. Joining 2-mm-thin sheets is an additional challenging task, which requires investigation. The feasibility of using FSW was assessed, and the effect of two critical FSW parameters, i.e., the weld tool geometry and weld speed, on the tensile strength of the joint was investigated. The welding tool geometry is the dominant parameter in FSW [39] as it affects the weld performance, and five different weld tool geometries (cylindrical tools with different pin geometries) were studied. Two of the tools followed the basic tool design with a single pin, each one of different pin diameter. The other three tools had radically different designs, two of which had a cylindrical pin, but one had a groove and the second one a protruding neck. The fifth design was one with a cylindrical pin which was eccentric to the axis of tool rotation. These experiments expand the earlier work performed by Dimopoulos et al. [7]. Similar work on the study of Al 7075 with these weld tools and FSW conditions, especially in thin (2 mm) sheets, has not yet been reported in the literature, so the current work expands the existing literature in this direction.

## 2. Materials and Methods

**Figure 1:**
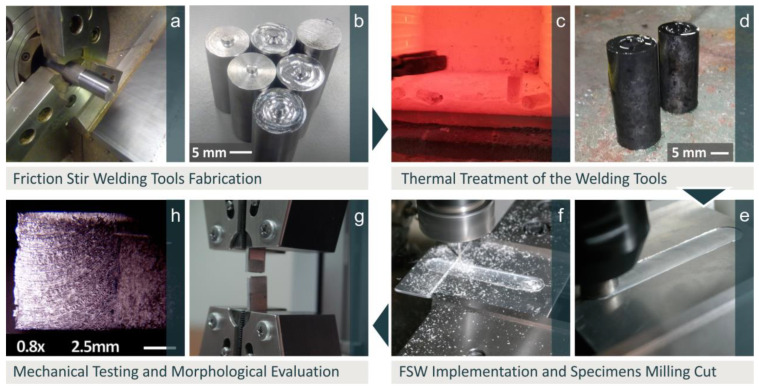
Procedure followed: (**a**,**b**) weld tool fabrication, (**c**,**d**) heat treatment of tools, (**e**,**f**) welding, (**g**,**h**) mechanical testing and morphological evaluation of welds.

Figure 1 shows the procedure followed, from the fabrication of weld tools (Figure 1a,b), heat treatment (Figure 1c,d), and welding of Al7075 sheets (Figure 1e,f), to the measurement of mechanical strength and the study of morphological characteristics of welds (Figure 1g,h).

**Figure 2:**
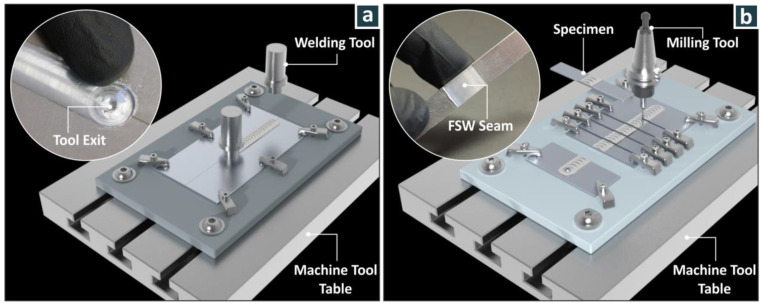
Experimental setup: (**a**) positioning the weld tool, (**b**) cutting the joint into tensile test specimens following welding.

The friction stir welding experiments were performed at room temperature (22 ± 1 °C) on a modified CNC milling machine (TM-1P Series Toolroom Mills CNC system of Haas, Oxnard, CA, USA), which was selected because it can withstand large forces and vibrations that are produced during welding. A specially designed fixture was prepared (designed and manufactured for the purposes of the work) to fix the thin (2 mm) aluminum sheets in the CNC machine for the implementation of the FSW process. The experimental setup is shown in Figure 2.

The welding tools were custom-made from WNr 1,6582/DIN 34CrNiMo6 steel rod of 16 mm diameter. This material is known for its hardness and can be heat treated. According to its technical datasheet, the density of the material is 7.84 gr/cm^3^, the tensile modulus of elasticity is 210 × 10^3^ MPa, its tensile strength (for this bar diameter) is 1180–1380 MPa, the elongation at fracture is 9%, the thermal conductivity is 37.7 W/m × K, and the specific heat capacity is 0.46 J/g × K. Its chemical composition is shown in Table 1. There were five different welding tools made. They were heat-treated in a furnace at 830 °C for 3.5 h to be oil quenched afterward [40].

**Figure 3:**
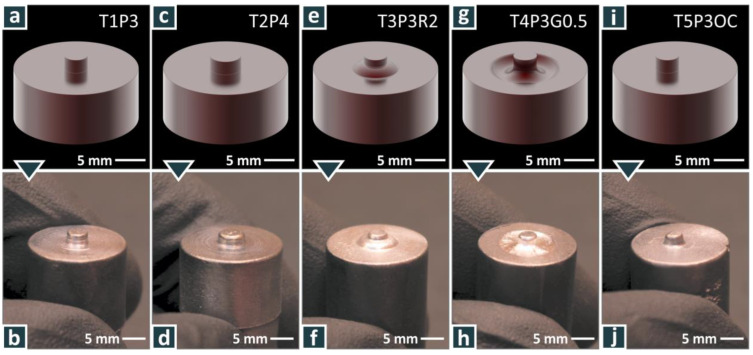
Five different welding tools: designs (**a**,**c**,**e**,**g**,**i**) and products (**b**,**d**,**f**,**h**,**j**).

All tools were of the same shoulder diameter of 16 mm, length of 50 mm, and pin height of 1.8 mm, but they differed in their pin diameter and shape, as shown in Table 2. The weld tool aimed to investigate the effect of the pin’s geometrical characteristics, without altering the cylindrical geometry. Photos of the designed and finished tools are shown in Figure 3a,b respectively. The weld tool rotational speed was kept constant at 1000 rpm in all cases studied, and it was chosen following preliminary tests and studies published in the literature. To investigate the effect of the welding speed on the weld performance, samples were joined with six different weld speeds, i.e., 80 mm/min, 110 mm/min, 150 mm/min, 200 mm/min, 250 mm/min, and 300 mm/min, with all the different weld tools produced. For each tool design, a weld was made at each welding speed.

**Figure 4:**
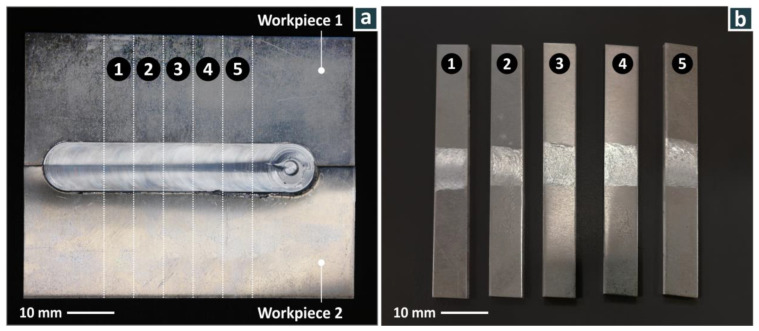
(**a**) Welded specimen with tensile test coupons. (**b**) Typical coupons for mechanical strength measurement after they were cut from the welded plate. Numbers indicate the number of each sample in the tests.

The aluminum alloy studied in the experiments was AA7075-0 (Table 3), with sheet dimensions of 100 mm × 40 mm × 2 mm. The AA7075-0 sheet was received in the non-heat-treated condition. The length of the weld was 75 mm, which was then cut into five smaller welded pieces with dimensions of 80 mm × 10 mm × 2 mm for tensile strength testing (Figure 4a). Because of welding thin sheets, both ends of the weld were distorted and had to have 25 mm cut off from each side (beginning and end of weld) to study the steady-state welds produced.

**Figure 5:**
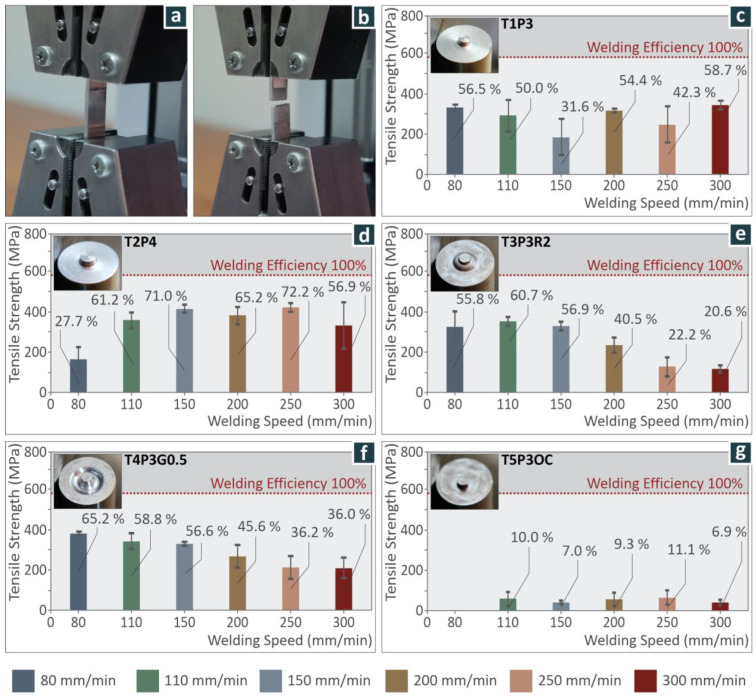
(**a**) Tensile test experimental setup, (**b**) welded specimen after it failed in the tensile test, and average tensile strength and deviation of welds produced with the tool (**c**) T1P3, (**d**) T2P4, (**e**) T3P3R2, (**f**) T4P3G0.5, and (**g**) T5P3OC. The welding efficiency is outlined in the graphs.

Welding was performed under various combinations of rotational and welding speed, to investigate the effect of tool design on mechanical strength. The tensile test was performed with an Imada MX2 (Northbrook, IL, USA) tension test machine, using grips of 40 mm at a 10 mm/min crosshead speed. Rectangular specimens were prepared from the welded aluminum sheets (Figure 4b). The tensile test specimens were not produced to a technical standard due to their size. For each setting of tool design and welding speed, five tensile tests were performed. The experimental setup for the tensile tests and a snapshot of a tensile test experiment on a sample after the failure of the sample are depicted in Figure 5a,b, respectively. An identical geometry to the welded samples was also prepared from the base material, the tensile strength was measured, and for comparison purposes, the weld efficiency, i.e., the ratio of the tensile strength of the weld to that of the base material, was calculated.

Tensile strength measurements were processed with statistical analysis tools and the main effects plots (MEP) and the interaction plots (IP) for the FSW parameters studied, i.e., the weld tool and the welding speed were compiled. In addition, the morphology of the fracture surface was evaluated under a microscope to identify the fracture mechanism and possible defects in the weld of the samples. A KERN OZR5 (KERN & SOHN GmbH, Balingen, Germany) stereoscope was used and images were acquired using a KERN ODC 832 5MP camera (KERN & SOHN GmbH, Balingen, Germany).

## 3. Results and Discussion

It was consistently found that the initial and final coupons from each welding sheet were of noticeably lower strength than the ones in the middle because of the sheet holding arrangement in a vice during welding. This allowed for distortion of the thin sheet specimens. To acquire accurate measurements of use to industry, the tensile strength measurements for these coupons were removed from the data.

It was also found that, at the times when the tool or welding speed was altered, the initial welding run was rarely successful, and most of the time did not produce a uniform weld along its length while it failed. This was associated with the low initial temperature of the tool. When the tool was used again, welds were sound as the tool had been heated from the initial run. However, if welding was to be repeated on the same sample for a second time, a small exit crack would form before lifting the tool. These issues relating to the first run can be resolved by pre-heating the tool. The development of exit cracks requires further study to address them properly.

In addition, if the groove of the tool T4P3G0.5 was filled with material, which was extruded during welding, the tensile strength results were affected negatively.

The first tool, T1P3, produces joints of improved strength at a welding speed of 300 mm/min, with a similar improvement at welding speeds of 80 mm/min and 200 mm/min. The three coupons show a small range of measurements in comparison to the other coupons, and the lowest strength is achieved at a welding speed of 150 mm/min (as shown in Figure 5c).

The second tool, T2P4, shows improved strength at welding speeds of 250 mm/min, 150 mm/min and 200 mm/min. The largest range of measurements in the mechanical strength measurements is at a welding speed of 300 mm/min, while the lowest strength is at a welding speed of 80 mm/min, as shown in Figure 5d.

The third tool, T3P3R2, shows improved mechanical strength measurements at welding speeds of 110 mm/min, 80 mm/min, and 150 mm/min. All mechanical strength measurements are consistent, with no large range of measurements in comparison to the other tool designs. It appears that the lowest mechanical strength achieved is at a welding speed of 300 mm/min, as shown in Figure 5e.

The fourth tool, T4P3G0.5, shows improved strength measurements at welding speeds of 80 mm/min, 110 mm/min, and 150 mm/min. The largest range of measurements in mechanical strength is at a welding speed of 300 mm/min, while the lowest strength measured is at a welding speed of 250 mm/min, as shown in Figure 5f.

The fifth tool, T5P3OC, shows improved mechanical strength at a welding speed of 250 mm/min, as well as at a welding speed of 110 mm/min, while the strength at 200 mm/min is lower. All these settings showed an appreciable range in mechanical strength measurements in comparison to the other tools. Finally, as shown in Figure 5g, the lowest mechanical strength measured is at a welding speed of 80 mm/min. The combination of this welding speed together with the off-center pin did not produce favorable conditions for a strong weld. It should be stated that this tool design produces the lowest mechanical strengths measured compared to others.

The weld efficiency (ratio of tensile strength of the weld to the base material) was also calculated. As expected, weld efficiency differs between welding tool designs. The highest weld efficiency was achieved with the T2P4 tool, while the worst weld efficiency was achieved with the T5P3OC tool, possibly due to the extensive material mixing effect of that design. The other three tools achieved rather similar results, with welding efficiencies in the range of about 40–65%, and an average weld efficiency of about 50%. The highest weld efficiency of 72.20% was achieved with T2P4 at a weld speed of 250 mm/min.

**Figure 6:**
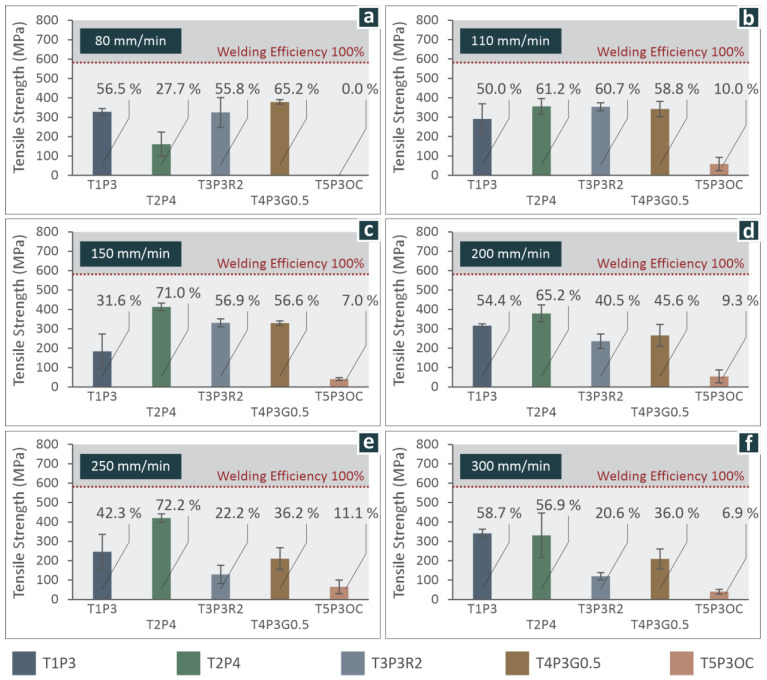
Average tensile strength at welding speeds of (**a**) 80 mm/min, (**b**) 110 mm/min, (**c**) 150 mm/min, (**d**) 200 mm/min, (**e**) 250 mm/min, and (**f**) 300 mm/min. The welding efficiency is noted.

When comparing the various tool designs, results in Figure 6a show that, at a welding speed of 80 mm/min, the highest strength is achieved with welds produced with tool T4P3G0.5, while tools T1P3 and T3P3R2 produce similar results, with the latter having a larger range of measurements. The tool design which failed, as it produced very poor results, was T5P3OC, possibly due to the extensive plastic material mixing that resulted, given the thickness of the sheets.

For welds performed at a welding speed of 110 mm/min (Figure 6b), the highest mechanical strength was achieved with tool T2P4, although close mechanical strengths were achieved with tools T3P3R2 and T4P3G0.5. The largest range of measurements in mechanical strength was measured with tool T1P3 compared to the others. Of all the tool designs, tool design T5P3OC again exhibited the lowest mechanical strength, as shown in Figure 6b.

For a welding speed of 150 mm/min (Figure 6c), the highest mechanical strength achieved was with tool T2P4, and close to that was with tools T3P3R2 and T4P3G0.5. None of the tool designs used had a large range in measurements. Again, tool T5P3OC showed the lowest mechanical strength, as depicted in Figure 6c.

For a welding speed of 200 mm/min (Figure 6d), the highest mechanical strength was achieved with tool T2P4, although it showed the largest range of values of mechanical strength measured at that setting, and tool T5P3OC achieved the lowest strength, as shown in Figure 6d.

For a welding speed of 250 mm/min (Figure 6e), the highest mechanical strength achieved was with tool T2P4. The mechanical strength achieved was much larger compared to the other tool designs, and tool design T5P3OC demonstrated the lowest mechanical strength and largest range of values measured, as shown in Figure 6e.

Finally, for a welding speed of 300 mm/min (Figure 6f), the largest mechanical strength achieved was for welds produced with tool T1P3, and similar to that was tool design T2P4, which showed a larger range in mechanical strengths measured. In addition, the mechanical strength of the welds produced with tool T4P3G0.5 had a large range in measurements. As before, tool T5P3OC showed the lowest mechanical strength, as depicted in Figure 6f.

The overall comparison shows that tool T2P4 produces welds with the highest mechanical strength under all welding settings studied. This tool design produced the best results at welding speeds of 250 mm/min and 150 mm/min. In addition, tool designs T3P3R2 and T4P3G0.5 show that at a lower welding speed, their tensile strength was high, but as the speed was increased, their tensile strength was affected negatively in a significant manner. The tool design T1P3 produced welds of inconsistent mechanical strength at various welding conditions, even though it was the best tool design at 300 mm/min welding speed. The tool design T5P3OC showed consistently poor mechanical strength at all welding conditions, with fracture stresses never exceeding 65 MPa. A 3D plot was made to compare the tensile strengths of the five welding tool designs at the welding speeds studied, as shown in Figure 9. These differences in the results between the different weld tools reflect the pronounced effect of tool geometry on mechanical strength. Figure 9 combines all these measurements. So, tool T2P4 produces poor welds at 80 mm/min, but in all other settings produces sound welds of great mechanical strength compared to the other tool designs.

**Figure 7:**
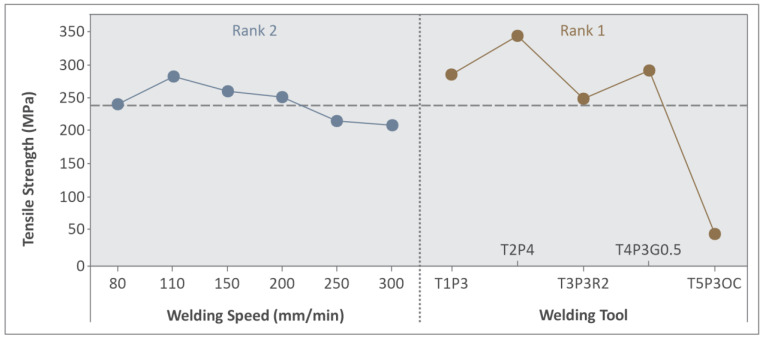
Main effect plots for the two FSW parameters studied, i.e., welding speed and welding tool.

**Figure 8:**
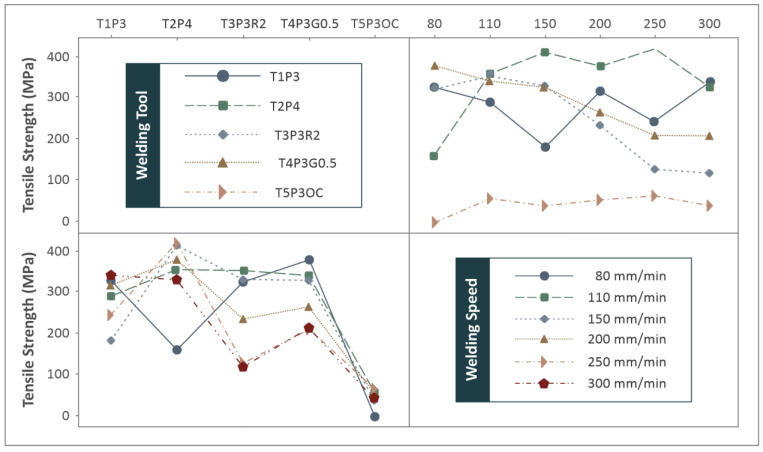
Interaction plots for the two FSW parameters studied, i.e., welding speed and welding tool.

**Figure 9:**
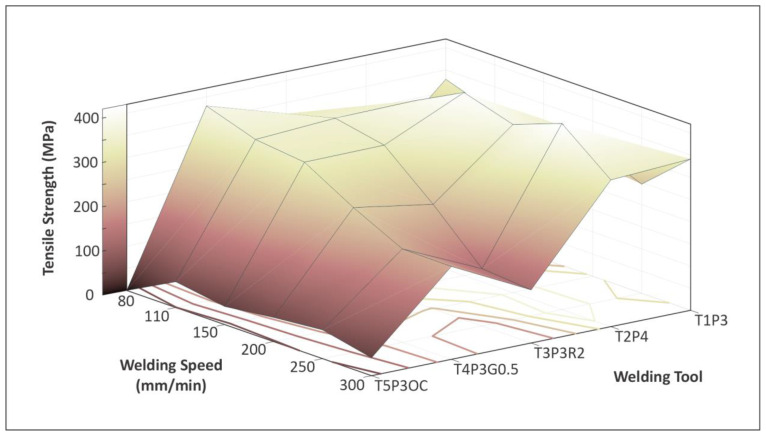
Mechanical strength measurements surface for different welding tools.

From the statistical analysis of the results, plots of the main effects were compiled for the welding speed and the weld tool (Figure 7). The welding speed does not significantly affect tensile strength and it is of lesser importance of the two parameters studied. Due to its effect, the weld tool is the dominant parameter in the work, with the highest tensile strength achieved with the T2P4 tool and the worst with the T5P3OC tool. The corresponding interaction plots are shown in Figure 8. Overall, no synergistic relations between parameters are observed in the plots. A rather similar pattern can be observed in the case of weld tools if the 80mm/min weld speed case is excluded. The remaining weld speeds show a rather synergistic response in this case. Figure 9 summarizes the tensile strength results compared to the welding speed and the weld tool in the 3D surface plot.

**Figure 10:**
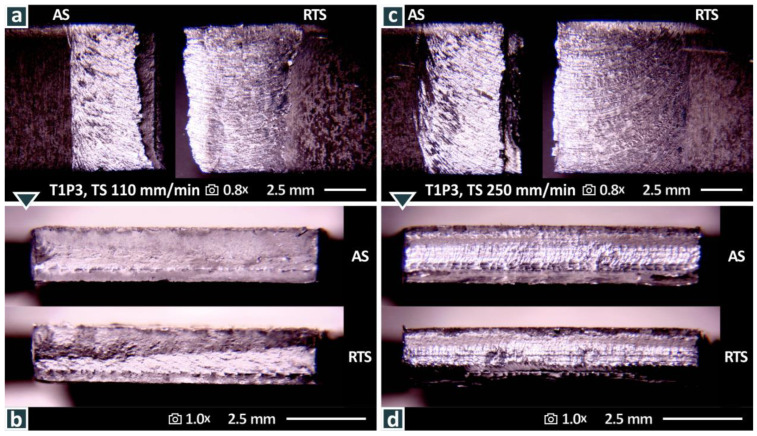
Stereoscopic microscopy images for the T1P3 weld tool at weld speeds of 110 mm/min: (**a**) top surface, (**b**) fracture surface; and 250 mm/min: (**c**) top surface, (**d**) fracture surface.

**Figure 11:**
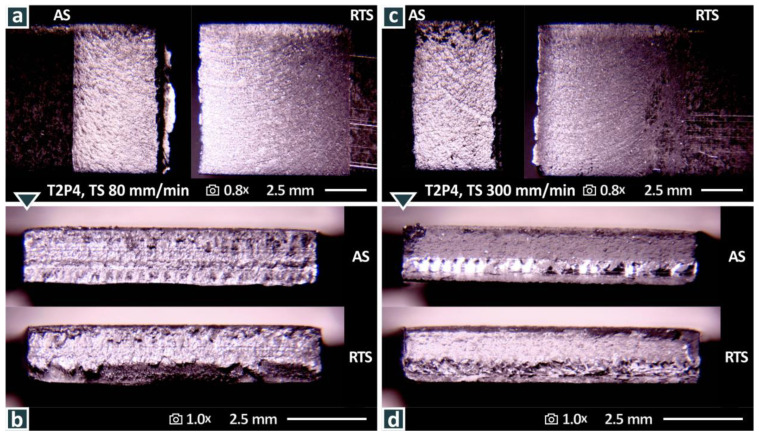
Stereoscopic microscopy images for the T2P4 weld tool at weld speeds of 80 mm/min: (**a**) top surface, (**b**) fracture surface; and 300 mm/min: (**c**) top surface, (**d**) fracture surface.

To study the morphology of the fractured welded specimens, images were acquired with a stereoscope (Figure 10). In Figure 10a, the top surface of a randomly selected specimen welded with the T1P3 tool at 110 mm/min is shown. The advancing side (AS) and the retreating side (RTS) are indicated in the figure. The nugget zone (NZ), the thermo-mechanically affected zone (TMAZ), and the heat-affected Zone HAZ) can also be distinguished in the figure. The fracture surface, as viewed from the top of the joint (Figure 10a,c and Figure 11a,c), shows a fracture at 45 degrees to the axis of tension where the maximum shear stress is resolved. The fracture surfaces appear uniform (linear) across the width in both cases, with failure occurring in the NZ. In both cases, the fracture surface is not in the middle of the NZ, but is closer to the AS. In both samples, no hook defects were observed in the fracture area.

**Figure 12:**
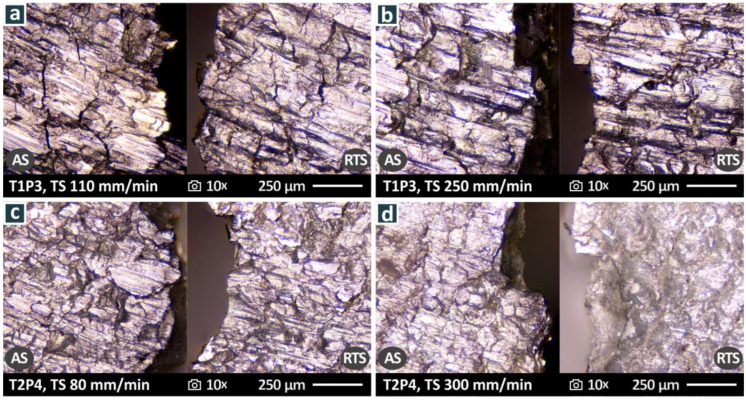
Higher magnification stereoscopic microscopy images for the T2P4 weld tool at weld speeds of 80 mm/min: (**a**) top surface, (**b**) fracture surface; and 300 mm/min: (**c**) top surface, (**d**) fracture surface.

Figure 11 shows images corresponding to Figure 10 for specimens welded again at two different weld speeds (Figure 11a,b—80 mm/min, Figure 11c,d—300 mm/min) with the T2P4 tool, where similar observations to Figure 10 can be made. The top surface of the samples is smoother compared to the samples welded with the T1P3 tool. In the sample welded at 80 mm/min, a material projection is observed at the AS on the bottom of the sample. Regarding the fracture surfaces, in the specimen that was welded at 80 mm/min (Figure 11b), the fracture surface is intensively rough, and cavities can be observed. The specimen that was welded at 300 mm/min (Figure 11d) shows a more solid fracture surface, without cavities and defects. On the bottom of the sample, the surface is rougher, but it does not show the abnormalities of the sample welded at 80 mm/min. These differences agree with the mechanical test results with the samples welded at 80 mm/min having overall inferior tensile strength to the samples welded at 300 mm/min with the T2P4 weld tool. To present the morphological characteristics described above more clearly, Figure 12 shows the same samples at higher magnification.

## 4. Conclusions

From the experiments performed, the following conclusions can be drawn:

- The tool geometry strongly affects the friction stir welding process, as well as the welding speed. The tool design T2P4, with the largest pin, achieved the highest tensile strength, while the tool design T5P3OC, whose pin is off-center, was very poor in terms of welds produced.
- When the tool or welding speed was changed, the first welding run was rarely successful, without a uniform seam, due to the low initial temperature of the tool.
- When the welding was repeated for a second time using the same sample, right before the tool was raised, a small exit crack would develop.
- In addition, if the groove of the tool design T4P3G0.5 was filled with material, tensile strength results would be negatively affected.
- A weld efficiency of 72.20% was achieved with the T2P4 weld tool at 250 mm/min.

## Figures and Tables

**Table 1:** Chemical composition (wt.%) of the welding tool material.

WNr 1.6582/DIN 34CrNiMo_6_—Ø16 mm							
C	Mn	Si	P	S	Cr	Mo	Ni
0.30–0.38	0.5–0.8	0.40 max.	0.025 max.	0.025 max.	1.3–1.7	0.15–0.30	1.3–1.7

**Table 2:** Tool design parameters (all tools were of cylindrical shape, had a shoulder diameter of Ø16 mm, a pin height of 18 mm and a tool length of 50 mm, and were run with a tool tilting angle).

Tool Number	1	2	3	4	5
Tool Name	T1P3	T2P4	T3P3R2	T4P3G0.5	T5P3OC
Pin diameter (mm) Geometry specifics	3 Cylindrical	4 Cylindrical	3 Protruding radius at the base of pin R = 2 mm	3 Groove R0.5	3 Pin off-center

**Table 3:** Chemical composition (wt.%) of 7075 aluminum alloy.

Al Alloy	Si	Fe	Cu	Mn	Mg	Cr	Ni	Zn	Ti	Ga	V	Al
7075	0.12	0.2	1.4	0.063	2.53	0.2	0.004	5.62	0.03	0.008	0.016	bal.

## Data Availability

The data presented in this study are available in the article.
